# Prevalence and risk factors of bowel symptoms in Korean patients with ulcerative colitis in endoscopic remission: a retrospective study

**DOI:** 10.1186/s12876-020-01597-1

**Published:** 2021-01-06

**Authors:** Kwangwoo Nam, Sang Hyoung Park, Jun Ho Oh, Ho-Su Lee, Soomin Noh, Jae Cheol Park, Jin Yong Kim, Eun Hye Oh, Jeongseok Kim, Nam Seok Ham, Sung Wook Hwang, Dong-Hoon Yang, Byong Duk Ye, Jeong-Sik Byeon, Seung-Jae Myung, Suk-Kyun Yang

**Affiliations:** 1 Department of Gastroenterology, Dankook University Hospital, Dankook University College of Medicine, Cheonan, Korea; 2 Department of Gastroenterology, Asan Medical Center, University of Ulsan College of Medicine, 88, Olympic-ro 43-gil, Songpa-gu, Seoul, 05505 Korea; 3 University of Ulsan College of Medicine, Seoul, Korea; 4 Department of Biochemistry and Molecular Biology, University of Ulsan College of Medicine, Seoul, Korea

**Keywords:** Patient-reported outcome, Bowel symptoms, Rectal bleeding, Stool frequency, Ulcerative colitis

## Abstract

**Background:**

Many patients with ulcerative colitis (UC) in clinical remission frequently complain of bowel symptoms such as increased stool frequency (SF) and rectal bleeding (RB). However, studies on these patient-reported outcomes in patients with inactive UC are limited, especially in Korea. Therefore, we investigated the prevalence and risk factors of bowel symptoms in Korean patients with inactive UC.

**Methods:**

We investigated the prevalence of bowel symptoms in patients with endoscopically quiescent UC between June 1989 and December 2016 using a well-characterized referral center-based cohort. The Mayo clinic score (MCS) was used to evaluate bowel symptoms at the most recent visit near the date of endoscopy. Clinical characteristics of the patients were compared based on the presence or absence of bowel symptoms.

**Results:**

Overall, 741 patients with endoscopically quiescent UC were identified, of whom 222 (30%) and 48 (6.5%) had an SF and RB subscore of ≥ 1, respectively. Patients with bowel symptoms (SF + RB ≥ 1; n = 244 [32.9%]) had higher rates of left-sided colitis (E2) or extensive colitis (E3) than patients without bowel symptoms (SF + RB = 0; n = 497 [67.1%]; *P* = 0.002). Multivariate analysis revealed that female sex (odds ratio [OR]: 1.568; 95% confidence interval [CI]: 1.023–2.402; *P* = 0.039) and E2 or E3 (OR 1.411; 95% CI 1.020–1.951; *P* = 0.038) were the significant risk factors for increased SF.

**Conclusions:**

This study revealed that one-third of patients with endoscopically quiescent UC reported increased SF. Female sex and disease extent may be associated with bowel symptoms.

## Background

Ulcerative colitis (UC) is a chronic idiopathic inflammatory disease involving colonic mucosa and submucosa. The incidence and prevalence of UC have significantly increased over the past decades in Europe (incidence: 57.9/100,000 person-years in Faroe Islands [2011]; prevalence: 505/100,000 in Norway [1990–1993]) and North America (incidence: 23.14/100,000 person-years in Canada [1996–2009]; prevalence: 286.3/100,000 in United States [2011]) [1, 2]. A similar trend has been observed in Asia (incidence: 4.6/100,000 person-years in Korea [2006–2012]; prevalence: 57.3/100,000 in Japan [2003–2005]), especially in Korea (incidence: 5.82/100,000 person-years [2011–2015], prevalence: 76.7/1000,000 [2015]) [2, 3]. Patients with UC typically experience periodic clinical remission and relapse [4–6]. However, many patients with UC who receive appropriate medical treatment can stay in remission with mild disease activity, and recent studies have reported that colectomy rates have significantly decreased among these patients [7–9].

Although the prime therapeutic target in patients with UC is endoscopic mucosal healing [10], a growing interest has been noted in evaluating patient-reported outcomes (PROs) regarding the disease activity. Stool frequency (SF) and rectal bleeding (RB) are commonly evaluated PROs. Mayo clinic score (MCS) and Truelove and Witt’s score, the most commonly used disease activity and severity indexes for UC, both include SF and RB. An increase in SF and presence of RB are considered composite indexes that suggest disease relapse before endoscopic confirmation [11, 12]. In addition, PROs that include SF and RB have presented good correlation with disease activity of UC [13].

Many patients with UC in clinical remission frequently complain of bowel symptoms such as increased SF and RB. In previous studies, irritable bowel-like symptoms were more commonly reported in patients with inflammatory bowel disease (IBD) with low disease activity (33%–46%) than in healthy controls (7%) or patients with irritable bowel syndrome (IBS; 8%) [14–16]. Previously, IBS was considered completely different from IBD; however, recent studies have suggested that some overlap may exist between these two disease entities [17]. Although the degrees of inflammation and visceral hypersensitivity between IBD and IBS are different, they have similarities regarding the brain-gut axis, some genetic factors, dysbiosis, and impaired epithelial barrier function [18].

To date, there have been few studies on symptom-based PROs in patients with inactive UC, especially among Korean patients [19]. Therefore, this study investigated the prevalence and risk factors of bowel symptoms in Korean patients with inactive UC.

## Methods

### Patients and study design

This study was based on a well-characterized referral center-based large cohort of Korean patients with UC. Medical records of patients enrolled in the Asan IBD registry between June 1989 and December 2016 were reviewed [7]. This registry includes all patients diagnosed with UC and treated at the Asan Medical Center. The diagnosis of UC was based on the conventional clinical, radiologic, endoscopic, and histopathologic criteria, as described previously [20]. Demographic and clinical features of the enrolled patients were evaluated. Immunosuppressive drugs used at any point in time were classified as follows: corticosteroids (orally or intravenously administered prednisolone or methylprednisolone; a daily dose equivalent to ≥ 20 mg of prednisolone for ≥ 4 weeks), immunosuppressants (azathioprine, 6-mercaptopurine, methotrexate, cyclosporine, or tacrolimus), and biologics (infliximab, adalimumab, golimumab, or vedolizumab).

### Endoscopic and clinical evaluation

Endoscopic evaluation was performed for the surveillance of UC after clinical remission. In case of multiple endoscopic evaluation, the most recent quiescent examination was selected for the evaluation. Endoscopic remission was defined as no evidence of active disease in colonoscopy or sigmoidoscopy (Mayo endoscopic subscore [MES] = 0). The maximum extent of UC was assessed based on previous colonoscopy findings and classified as proctitis (E1), left-sided colitis (E2), and extensive colitis (E3). MCS was obtained at each routine visit to evaluate clinical status [7, 21]. In our center, partial MCS (MCS without MES) of each patient was routinely determined at their every visit. The presence of bowel symptoms was evaluated using SF and RB from MCS at the visit near the date of endoscopy. Patients were classified based on the presence or absence of bowel symptoms (SF + RB ≥ 1 vs. SF + RB = 0), and the clinical characteristics of each group were compared. These findings were used to evaluate the risk factors of bowel symptoms in patients with inactive UC.

### Statistical analysis

Continuous variables were expressed as medians with interquartile range (IQR) and were compared using the Mann–Whitney U–test. Categorical variables were expressed as numbers with percentages and were compared using the chi-square test or Fisher’s exact test. Logistic regression analysis was performed to evaluate the risk factors of bowel symptoms in patients with inactive UC. Variables with *P* < 0.1 in univariate analyses were further evaluated using multivariate analysis. All statistical analyses were performed using SPSS software version 25.0 (SPSS Inc., Chicago, IL). *P* < 0.05 was considered statistically significant.

## Results

### Demographics and baseline characteristics of the patients

**Fig. 1:**
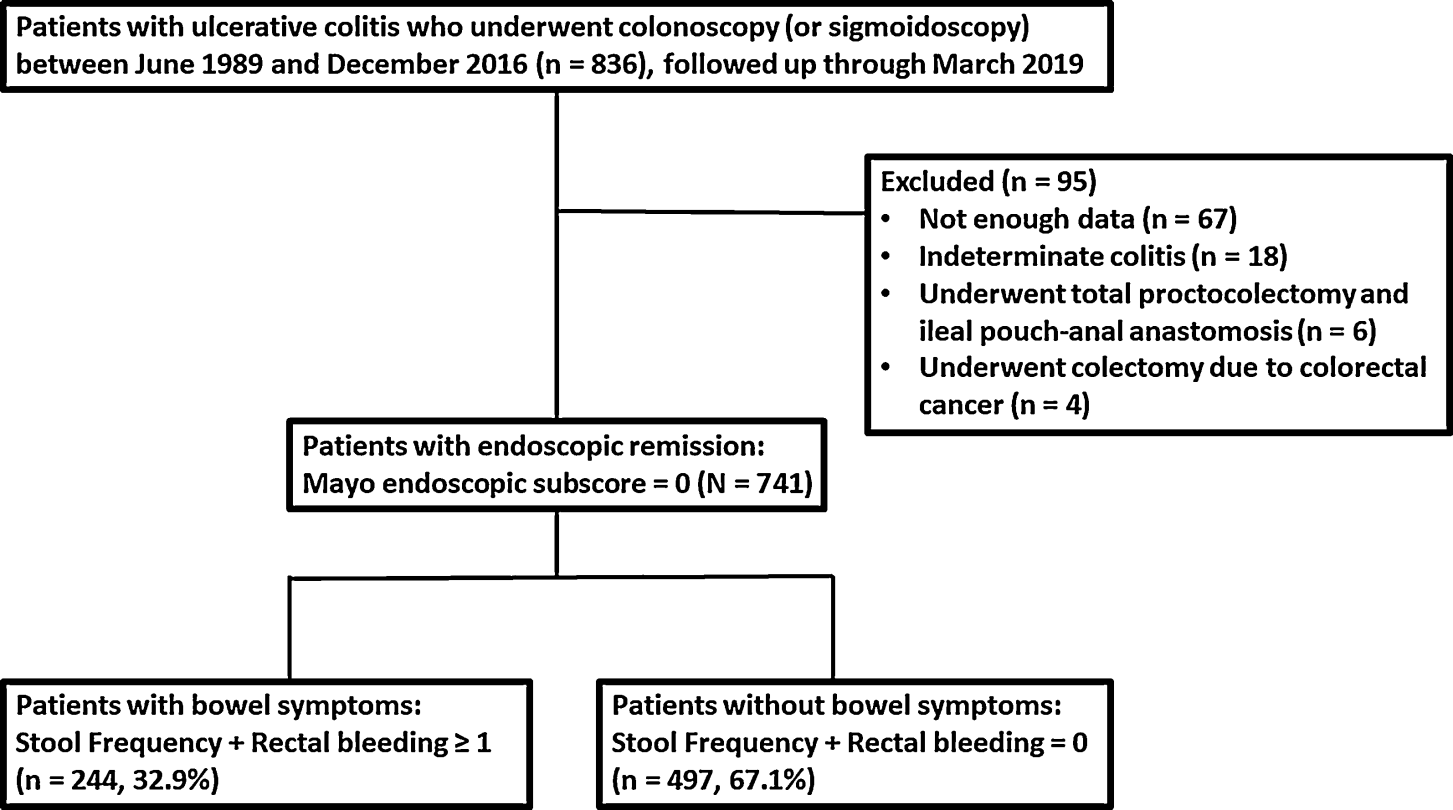
Study flowchart

A total of 741 patients were confirmed with at least one or more endoscopically quiescent UC (MES = 0) between June 1989 and December 2016. These patients were followed up until March 2019 (Fig. 1). The total patient-years of follow-up was 8875.3 years, and the median patient-years of follow-up per patient was 11.3 years (IQR 7.4–16.3). The median age at the time of UC diagnosis was 41 years (IQR 32–51). In total, 343 (46.3%) patients were women. The analysis of maximum extent of UC revealed that E3 was the most common (39.1%), followed by E2 (32.4%) and E1 (26.6%). Patients’ history of immunosuppressive drug use was found to be as follows: corticosteroids, 57.1%; immunosuppressants, 25.8%; and biologics, 10.7% (Table 1).

**Table 1:** Demographic and clinical features of the studied patients

	Total (N = 741)
*Total patient-years of follow-up (years)*	8875.3
Median (IQR) (years)	11.3 (7.4–16.3)
*Demographic features*	
Age at diagnosis of UC, median (IQR) (years)	41.0 (32–51)
Sex, female, n (%)	343 (46.3)
Family history of IBD, n (%)	68 (9.2)
History of appendectomy	20 (2.7)
Current or ex-smoker	299 (40.4)
Extraintestinal manifestation, n (%)	21 (2.8)
PSC, n (%)	9 (1.2)
*Maximum extent of UC*	
Proctitis (E1), n (%)	197 (26.6)
Left-sided colitis (E2), n (%)	240 (32.4)
Extensive colitis (E3), n (%)	290 (39.1)
Not enough data, n (%)	4 (0.5)
*History of immunosuppressive drug use for IBD*	
CS (prednisolone or methylprednisolone), n (%)	423 (57.1)
IS (azathioprine/6-mercaptopurine, methotrexate, cyclosporine, or tacrolimus), n (%)	191 (25.8)
Biologics (infliximab, adalimumab, golimumab, or vedolizumab), n (%)	79 (10.7)

CS, corticosteroid; IBD, inflammatory bowel disease; IQR, interquartile range; IS, immunosuppressant; PSC, primary sclerosing cholangitis; UC, ulcerative colitis

### Characteristics of patients with bowel symptoms and comparison with patients without bowel symptoms

Medical records of the enrolled patients were reviewed and patients with bowel symptoms (SF + RB ≥ 1; n = 244 [32.9%]) and without bowel symptoms (SF + RB = 0; n = 497 [67.1%]) were identified (Fig. 1). Interval between the date of endoscopy and the nearest visit at evaluation was a median of 8 days (IQR: 6.5–15). No significant differences were noted between the two groups regarding demographic features and patient-years of follow-up. The analysis of maximum extent of UC revealed that E2 or E3 were more common among patients with bowel symptoms than among those without bowel symptoms (*P* = 0.002). With regard to the history of immunosuppressive drug use, patients with bowel symptoms were more likely to have a history of corticosteroid (*P* < 0.001) and immunosuppressant use (*P* = 0.030) than those without bowel symptoms (Table 2).

**Table 2:** Comparison of demographic and clinical characteristics of the patients with endoscopically quiescent ulcerative colitis with bowel symptoms with those without bowel symptoms

	UC patients with bowel symptoms (n = 244)	UC patients without bowel symptoms (n = 497)	*P* value
*Total patient-years of follow-up (years)*	2970.6	5904.7	0.489*
Median (IQR) (years)	11.6 (6.3–15.4)	10.8 (6.3–15.4)	
*Demographic features*			
Age at diagnosis of UC, median (IQR) (years)	41 (31–52)	41 (32–51)	0.680*
Sex, female, n (%)	101 (41.4)	242 (48.7)	0.061**
Family history of IBD, n (%)	22 (9.0)	46 (9.3)	0.916**
History of appendectomy, n (%)	6 (2.5)	14 (2.8)	0.778**
Current or ex-smoker, n (%)	90 (36.9)	209 (42.1)	0.178**
Extraintestinal manifestation, n (%)	4 (1.6)	17 (3.4)	0.170**
PSC, n (%)	3 (1.2)	6 (1.2)	1.000***
*Maximum extent of UC*			0.002***
Proctitis (E1), n (%)	44 (18.0)	153 (30.8)	
Left-sided colitis (E2), n (%)`	86 (35.2)	154 (31.0)	
Extensive colitis (E3), n (%)	108 (44.3)	182 (36.6)	
Not enough data, n (%)	6 (2.5)	8 (1.6)	
*History of immunosuppressive drug use for UC, n (%)*			
CS (prednisolone or methylprednisolone), n (%)	**160 (65.6)**	**263 (52.9)**	< 0.001**
IS (azathiopurine/6-mercaptopurine, methotrexate, cyclosporine, or tacrolimus), n (%)	**75 (30.7)**	**116 (23.3)**	0.030**
Biologics (infliximab, adalimumab, golimumab, or vedolizumab), n (%)	23 (9.4)	56 (11.3)	0.445**

Bold font indicates statistical significance

CS, corticosteroid; IBD, inflammatory bowel disease; IQR, interquartile range; IS, immunosuppressant; PSC, primary sclerosing cholangitis; UC, ulcerative colitis

^*^Mann–Whitney test, **chi-square test, ***Fisher’s exact test

Among the patients with bowel symptoms (n = 244), 222 (91.0%) patients had SF ≥ 1, whereas 196 (80.3%) patients had RB = 0. The distribution of SF + RB was as follows; SF + RB = 1, 191 (78.3%) patients; SF + RB = 2, 36 (14.8%) patients; SF + RB = 3, 14 (5.7%) patients; SF + RB = 4, 2 (0.8%) patients; and SF + RB = 5, 1 (0.4%) patient (Table 3).

**Table 3:** Distribution of partial Mayo clinic score in patients with bowel symptoms and in those without bowel symptoms

	UC patients with bowel symptoms (n = 244)	UC patients without bowel symptoms (n = 497)
*Stool frequency subscore at evaluation, n (%)*		
0	22 (9.0)	497 (100)
1	192 (78.7)	0 (0)
2	25 (10.2)	0 (0)
3	5 (2.0)	0 (0)
*Rectal bleeding subscore at evaluation, n (%)*		
0	196 (80.3)	497 (100)
1	39 (16.0)	0 (0)
2	5 (2.0)	0 (0)
3	4 (1.6)	0 (0)
*Stool frequency + rectal bleeding subscore at evaluation, n (%)*		
0	0 (0)	497 (100)
1	191 (78.3)	0 (0)
2	36 (14.8)	0 (0)
3	14 (5.7)	0 (0)
4	2 (0.8)	0 (0)
5	1 (0.4)	0 (0)

UC, ulcerative colitis

### Risk factors of bowel symptoms in patients with endoscopically quiescent UC

To identify the risk factors of bowel symptoms in patients with endoscopically quiescent UC, we performed logistic regression analysis. In the univariate analysis, female sex, E2 or E3 in maximum extent, a history of corticosteroid use, and a history of immunosuppressant use showed *P* < 0.1 for both increased SF and SF + RB. In the multivariate analysis, female sex (odds ratio [OR]: 1.568; 95% confidence interval [CI]: 1.023–2.402; *P* = 0.039) and E2 or E3 in maximum extent (OR: 1.411; 95% CI: 1.020–1.951; *P* = 0.038) were found to be the significant risk factors of increased SF. In addition, E2 or E3 in maximum extent (OR: 1.508; 95% CI: 1.002–2.268; *P* = 0.049) was the significant risk factor of SF + RB (Table 4).

**Table 4:** Factors associated with bowel symptoms in patients with endoscopically quiescent ulcerative colitis. (A) stool frequency, (B) stool frequency + rectal bleeding

	OR	95% CI	*P* value
*(A) Stool frequency*			
Sex, female	**1.568**	**1.023–2.402**	**0.039**
Maximum extent of UC, left-sided colitis or extensive colitis	**1.411**	**1.020–1.951**	**0.038**
History of CS use	1.348	0.906–2.006	0.140
History of IS use	1.243	0.834–1.850	0.186
*(B) Stool frequency* + *rectal bleeding*			
Sex, female	1.315	0.960–1.800	0.088
Maximum extent of UC, left-sided colitis or extensive colitis	**1.508**	**1.002–2.268**	**0.049**
History of CS use	1.366	0.930–2.007	0.062
History of IS use	1.117	0.756–1.652	0.578

Bold font indicates statistical significance

CI, confidence interval; CS, corticosteroid; IS, immunosuppressant; OR, odds ratio

## Discussion

We evaluated bowel symptoms in patients with endoscopically quiescent UC by using PROs based on SF and RB derived from MCS and found that approximately one-third of patients complained of bowel symptoms mainly due to increased SF. The multivariate analysis revealed that female sex and greater extent of bowel damage (E2 or E3) were significant risk factors for increased SF and greater extent of bowel damage for SF + RB.

Monitoring disease activity in patients with UC is essential. MCS that includes SF, RB, MES, and PGA is most commonly used as the disease activity index. Because frequent endoscopic evaluation in patients with inactive UC is not usually performed, partial MCS is more commonly used during follow-up visits in the real-world setting. In general, PGA may reflect patients’ impaired quality of life due to abdominal pain, discomfort; however, due to the subjective aspects of PGA, it is difficult to consider MCS as an objective index of PRO. In contrast, SF and RB are symptom-based PROs that are usually considered clinical targets of remission [22] and are associated with endoscopic remission. Studies have reported that in some patients, these PROs did not exactly correlate with endoscopic findings, and up to one third of the patients with endoscopically and histologically inactive UC may experience increased SF [23]. In the present study, we defined strict endoscopic remission as MES = 0, and 33% of the patients still complained of increased SF and/or RB, mostly due to increased SF (30%).

There have been some suggestions regarding PROs in patients with UC. Walmsley et al. suggested a simple clinical colitis activity index including bowel frequency (day and night), urgency, bloody stool, general well-being, and extracolonic features had good correlation with other complex indexes [24]. Bewtra et al. indicated that SF, RB, and patient-reported general well-being accurately determined clinical disease activity [13]. SF and RB can be used in routine clinical practice because they can easily derived from MCS. However, SF and RB in patients with endoscopically quiescent UC may present different patterns. Jharap et al. investigated the relationship between mucosal healing and PROs (SF + RB) in patients with UC who were treated with adalimumab or placebo, and reported that among the patients with MES = 0, the proportion of patients with SF ≥ 1 (71.2%) was higher than that of patients with RB ≥ 1 (12.8%) [25], which is consistent with our results (SF ≥ 1 vs. RB ≥ 1; 30% vs. 6.5%). This suggests that RB is more influenced by mucosal healing than SF, and other factors might be associated with increased SF in these patients. In addition, although 20% of patients with bowel symptoms showed RB ≥ 1 in this study, their rectal bleeding symptoms were not severe enough to cause anemia or necessitate red blood cell transfusion.

One possible explanation is that these patients might still have low-grade inflammation in the bowel wall, which was insufficient to generate definite erythema, erosion, or ulcers, but could provoke IBS-like symptoms. Low-grade inflammation may be associated with altered enteric nervous system and microbiota, similar to IBS. However, some discrepancies were reported between IBS-like symptoms and fecal calprotectin levels in patients with UC [17]. In the present study, female sex was a significant risk factor of increased SF, similar to IBS. In general, functional gastrointestinal disorders, including IBS, are more common in women than men [26, 27]. These gender difference is considered to be associated with the difference in visceral pain perception, autonomic function, and effects of the sex hormones. Thus, patients with endoscopically quiescent UC who present bowel symptoms may share common features with IBS than expected. Furthermore, management similar to that of IBS, such as low FODMAP diet and usage of bowel movement drugs, may be also helpful in these patients.

Previous disease extent can be another explanation. In the present study, the maximum extent of UC was analyzed, and it was found that E2 and E3 were more common in patients with bowel symptoms than in those without bowel symptoms (Table 2). Proximal disease extension of UC not only indicates the progression of UC but also a more damaged bowel. Long-term disease involvement of UC could lead to anatomical changes in the diseased bowel, which may be associated with impaired motility and absorptive function, similar to IBS [28]. In multivariate analysis, previous E2 or E3 suggesting greater bowel damage was a significant risk factor of increased SF and SF + RB. Henriksen et al. conducted a long-term follow-up study for 20 years on the prevalence of IBS-like symptoms in patients with UC and reported that the overall prevalence of IBS-like symptoms was 27%, which was not significantly different than those among patients with ongoing inflammation and those without signs of inflammation (25%–35%) [29]. These IBS-like symptoms might be affected by previous long-term bowel damage during 20 years.

This study has some limitations. First, the retrospective nature of this study cannot eliminate selection bias. Our study was based on a tertiary center-based registry, and hence, we could not avoid referral bias. Our results may not reflect the general aspect of bowel symptoms in patients with inactive UC. In addition, some patients’ drug histories were incomplete; thus we could not provide concomitant medication for UC treatment or bowel movement (e.g., anti-diarrheal drugs, laxatives). Second, we did not assess histological findings of endoscopically quiescent UC. The presence of histologic bowel inflammation in the absence of endoscopic activity may be the cause of bowel symptoms [17]. Fecal calprotectin levels, which correlate with mucosal activity, were available in Korean hospitals only after 2015. Thus, in this study, fecal calprotectin levels were not assessed in the majority of the enrolled patients and we could not use fecal calprotectin testing to evaluate disease activity in all enrolled patients. To overcome these limitations, we applied more strict criteria of endoscopic remission (MES = 0) than those applied in previous studies (MES = 0 or 1) [23, 29]. Third, we did not evaluate the relapse rate according to the patients’ bowel symptoms because we focused on temporal findings of bowel symptoms. In addition, MCS records bowel symptoms during previous 3 days by definition, thus it may not correctly reflect persistent bowel symptom. To minimize ovelap of temporal worsening of symptoms such as infectious enteritis, we minimize the interval between the day of endoscopy and the nearest visit (median 8 days). Fourth, some patients (2.2%) underwent sigmoidoscopy only after endoscopic remission; thus it is impossible to reveal possible residual inflammation in the proximal colon. However, these patients were in clinically stable state without evidence of disease aggravation; thus we included these patients. Fifth, most patients (84.4%) underwent endoscopy before the clinic visit; hence, endoscopy and relevant bowel preparation may provoke bowel symptoms in these patients [30].

## Conclusion

In conclusion, even in patients with endoscopically quiescent UC, approximately one-third of the patients reported bowel symptoms, especially increased SF. These results might be associated with female sex and previous significant bowel damage. Physicians who treat IBD patients must know these phenomena and could suggest proper management of bowel symptoms in patients with long-standing UC.

## Data Availability

The datasets used and/or analyzed during the crreunt study are available from the corresponding author or request.

## Abbreviations

CI: Confidence interval
IBD: Inflammatory bowel disease
IBS: Irritable bowel syndrome
IQR: Interquartile range
MCS: Mayo clinic score
MES: Mayo endoscopic subscore
OR: Odds ratio
PRO: Patient-reported outcome
RB: Rectal bleeding
SF: Stool frequency
UC: Ulcerative colitis
